# Parents’ Perception of Their 2–10-Year-Old Children’s Contribution to The Dyadic Parent-Child Relationship in Terms of Positive and Negative Behaviors

**DOI:** 10.3390/ijerph16071123

**Published:** 2019-03-28

**Authors:** Wolfgang Briegel, Jan Greuel, Sanna Stroth, Nina Heinrichs

**Affiliations:** 1 Department of Child and Adolescent Psychiatry, Psychosomatics and Psychotherapy, Leopoldina Hospital, 97422 Schweinfurt, Germany; 2 Department of Child and Adolescent Psychiatry, Psychosomatics and Psychotherapy, University of Würzburg, 97080 Würzburg, Germany; 3 Department of Psychology, Technical University of Braunschweig, 38106 Braunschweig, Germany; j.greuel@tu-braunschweig.de (J.G.); nheinric@uni-bremen.de (N.H.); 4 Department of Child and Adolescent Psychiatry, Psychosomatics and Psychotherapy, Philipps University, 35033 Marburg, Germany; stroth@staff.uni-marburg.de; 5 Department of Psychology, University of Bremen, 28359 Bremen, Germany

**Keywords:** parent-child relationship, child behavior, parental perception, inventory

## Abstract

Parent-child relationship is developed and changed through reciprocal interactions between a child and his/her parent, and these interactions can strongly influence the child’s development across domains (e.g., emotional, physical, and intellectual). However, little is known about the parental perception of the child’s contribution to the dyadic parent-child relationship in terms of positive and negative behaviors. We therefore aimed to develop and validate an economical parent-report instrument to assess these important aspects. The validation study included 1642 mothers (M_age_ = 37.1) and 1068 fathers (M_age_ = 40.4) of 1712 children aged 2–10 years (M_age_ = 6.6) who completed the new instrument, the Child Relationship Behavior Inventory (CRBI). Statistical results indicated that the CRBI is a reliable and valid measure. Mothers reported more positive child behaviors towards them, whereas fathers perceived fewer problems with problematic relationship behavior than mothers. In their parents’ perception, girls showed more positive and less problematic relationship behaviors than boys. The frequency of problematic child relationship behavior significantly decreased with increasing child age while positive relationship behavior did not show any correlation with the child’s age. To assess both positive and negative child relationship behaviors could be helpful to better understand the relevance of these different aspects for the development of the parent-child relationship.

## 1. Introduction

The parent-child relationship can be described as a complex, dynamic and unique dyadic system that is more than the sum of its parts [1,2]. It is characterized as non-voluntary, determined by laws and norms, interdependent, and transcontextual [1,3,4,5]. Parent-child relationship is developed and changed through daily, reciprocal interactions between a child and a parent in different relationship domains (e.g., attachment and authority) which form the basis for further interactions [1,6]. These interactions are able to strongly influence children’s development across domains (e.g., emotional, physical, and intellectual) [7]. The importance of the parent-child relationship to optimal child outcomes and to mental well-being over the lifespan has been well established across cultures and socio-economic levels [8].

Children are actively contributing to the relationship with their parents, and “both parent and child affect and are affected by the other’s behavior in a given interaction and across time in the relationship” ([1], p. 330). The contributions of a child to the relationship with the parent can be described in terms of usually positively or negatively evaluated behaviors, e.g., hugging or hitting a parent. There is clear evidence that positive (i.e., cooperative/prosocial) behaviors of children aged 2–12 years are more likely to provoke positive parental reactions, whereas children with negative (e.g., disruptive) behaviors often get negative reactions from their parents [9,10]. Parent reports also indicate that child behaviors which preserve the vertical nature of the relationship (e.g., compliance with parent requests) strengthen the parent-child relationship, whereas child behaviors that challenge or dismiss the parent’s authority (e.g., non-compliance) create tension [1].

With regard to child behaviors, gender specific differences are well documented. Compared to males, females aged 3 to 12 years have been found to be more concerned about sharing, turn-taking and cooperation, and to be more prosocial from childhood through adolescence [11,12], whereas boys present more disruptive behavior problems than girls during childhood and adolescence [13,14]. Additionally, child behavior seems to differ in interactions with mothers versus fathers [15] with both normal and hyperactive boys showing more negative interactions with mothers than fathers [16]. Moreover, children seem to show more compliance with fathers than mothers [17,18]. With mothers typically being the primary caretakers in families with traditional parenting roles [19], it is unclear to what extent differences between mothers and fathers are due to gender or caregiving aspects (e.g., primary versus secondary caregiver). Current social changes in family settings (e.g., same-sex parenting, fathers being primary caregivers) might help to unravel these confounding effects. Taking findings of different child interactions with mothers versus fathers into account, it is not surprising that, according to some studies, mothers tend to report more behavior problems in their children than fathers [18,20]. However, other studies did not find such differences between parents [21].

Given the fact that boys show more disruptive behavior problems than girls [13,14], one might expect boys to be more likely to elicit controlling behavior from parents. However, a recent meta-analysis revealed minimal differences in parental control of boys and girls [22]. With regard to gender effects on parents’ talk to their children, another meta-analysis found mothers to talk more and to use more supportive speech than fathers do [23]. In line with that, Tiano et al. [15] reported that mothers provided more positive and descriptive verbalizations than fathers. Interestingly, mothers talk more and use more supportive speech with daughters than with sons [23].

Positive and negative child behaviors towards parents and their relationship can promote or complicate the development of a positive parent-child relationship by influencing the parents’ perceptions of the child. Particularly, the effects of positive child behaviors on children’s relationships with their parents have been understudied [24]. Thus, a problem-focused approach does not provide a holistic understanding of children and their relationships [19]. Moreover, it can negatively affect families [25]. In contrast, the assessment of both strengths and difficulties can have several benefits. Among other aspects, it can increase the acceptability of the assessment process, positively impact client-therapist rapport, redirect caregivers from focusing solely on the child’s symptoms or behavior problems and guide treatment and prevention approaches [25]. Today, it is unclear in what way positive and negative child behaviors contribute to overall parent-child relationship quality.

Several instruments have been developed in the past to assess special dimensions of parent-child relationship, some of them focusing on the child (e.g., attachment or child behavior), and others focusing on parents (e.g., parenting style or emotional availability), among them being observational approaches, questionnaires, scales and checklists. Some of the best established observational instruments in the field are: the Strange Situation Procedure (SSP) [26], the Dyadic Parent-Child Interaction Coding System (DPICS) [27] and the Emotional Availability Scales (EAS) [28]. They are validated tools which cover different aspects of parent-child relationships, but all of them share a common disadvantage: they require intensive rater training. More economical with respect to cost, effort, and time are questionnaires regarding the parent-child relationship, which comprise parent self-reports (e.g., the Parenting Scale [29] and the Parent-Child Relationship Inventory (PCRI) [30]), parent reports on child behaviors (e.g., the Child Behavior Checklist (CBCL) [31] and the Eyberg Child Behavior Inventory (ECBI) [32]), and external rating scales (e.g., the Parenting Style Inventory (PSI-I) [33]). As parts of the Diagnostic Classification of Mental Health and Developmental Disorders of Infancy and Early Childhood-Revised Edition (DC: 0-3R) [34], the Parent-Infant Relationship Global Assessment Scale [PIR-GAS] and the Relationship Problems Checklist [RPCL] are tools for clinicians to evaluate the parent-child relationship on axis II.

Despite this variety of instruments to assess special aspects of parent-child relationship, to the authors’ best knowledge there is no valid and economical assessment instrument thus far to evaluate how, according to the parent’s perception, the child contributes to his/her relationship with the parent in terms of positive (e.g., hugging a parent) as well as negative behaviors (e.g., telling that he/she does not like the parent) towards the parent. However, the parents’ perception of their children’s behaviors towards them is a specific and important aspect impacting parent-child relationship. This perception can vary in the parent-perceived frequency of specific child behaviors (regardless of the objectively-measured frequency) as well as in the level of satisfaction or concern (despite similar frequencies) with such behaviors [35]. These perceptions guide parental decisions (e.g., whether to seek help for the child or not) and actions which in turn impact their children’s psychosocial development [36]. Moreover, parental perceptions and child characteristics have been found to influence one another [37,38].

Usually, parental perceptions include an objective component, referring to actual child characteristics, and a subjective component, comprising parent-related factors [36]. Interpretations and definitions of a child’s behavior as well as interpersonal attributions (i.e., attributions for another’s behavior) [39] play an important role in affecting how a parent behaves towards her or his child. Interpersonal attributions can be broadly subdivided into causal and responsibility attributions with causal attributions comprising internal and external attributions [40]. An internal attribution refers to parental perceptions of a child’s behavior as dispositional and intentional, whereas an external attribution refers to parental perceptions of a child’s behavior as situated in context, transitory, and even accidental [41]. This is in line with research on adult partner relationships showing a robust association between attributions, behavior and relationship quality [42,43]. Interestingly, it has been well demonstrated that parents of children without behavior problems “typically credit internal child factors (e.g., ability, effort) as the causes of positive behaviors, such as altruism, and excuse negative behaviors, such as norm violations, as due to external, transient, or uncontrollable causes.” ([44], p. 473). In summary, parental perceptions of their children are very important regardless of whether they are congruent or not with results from more objective sources of information [36].

As studies from the field of couple relationships have found support for a ratio model which suggests that what is predicting satisfaction among stable couples is the relative amount of positive to negative affect [45,46,47], it might also be that a ratio of positive to negative child behaviors towards their parents (and associated parental affects) might predict best parental satisfaction with the parent-child relationship. Furthermore, Gottman and Levenson investigated longitudinally what predicts the negative change (decline) of affective couple interaction [48]. They compared a set of variables as predictors (e.g., cognitions about the relationship, physiology) and concluded that the “most powerful model in predicting change” occurred to be the balance ratio model, which was based on the ratio of positivity to negativity. The ratio model is based on the assumption that negative affect is endemic in relationships regardless of the relationship quality and that this relationship will work to the extent that this negative affect is balanced. For couple relationships, Gottman stated that a ratio of 5:1 (positivity to negativity) was typical for stable marriages while 0.8:1 was indicative of unstable marriages [46]. Although parent-child relationship is asymmetric by nature and its comparison to marital relationship has many limitations (e.g., stability of the relationship), there are a number of similarities between partner-partner and parent-child relationship functioning (e.g., escalation traps, expectation of reciprocity). Therefore, we assumed that such a balance ratio could also be of high interest with regard to the parent-child relationship and its change over time.

In this project, we therefore aimed to develop and validate an economical parent-report instrument to assess the parental perception of 2- to 10-year-old children’s contributions to the dyadic parent-child relationship in terms of positive and negative child behaviors that allows the determination of a ratio for each dyad. Furthermore, we were interested in empirically exploring for the first time a ratio that might be predictive of stable and positive parent-child relationships. We conducted two studies to reach these goals. For the first study a multistep approach was used to develop a pre-trial questionnaire which afterwards underwent initial validation comprising internal consistency and exploratory factor analysis. With the second study, we aimed to collect data for a more comprehensive examination of the final version of the inventory. Based on the results of the first study, we planned to compute confirmatory factor analysis, internal consistency, item selectivity, inter-correlations of inventory scales, interrater-reliability, child gender and age effects, divergent and convergent as well as discriminative validity.

## 2. Materials and Methods

### 2.1. Study 1: Development and Initial Validation of the Child Relationship Behavior Inventory

To develop the inventory, a multistep approach was administered following guidelines on the development of psychological tests [49,50]. For the initial item generation, a systematic literature research on the topic in MEDLINE (via PubMed) and PSYNDEX was conducted. We found several instruments for assessing related aspects of parent-child relationship (see above). Thus, an item-pool was drafted based on observations of interactions between children aged 2–10 years and their parents in everyday situations and interviews with parents of children of that age group. Additionally, for the generation of items referring to negative child behaviors towards a parent, parent reports on general behavior problems (across relationships and situations) like the Child Behavior Checklist [31] and the Eyberg Child Behavior Inventory (ECBI) [32] were taken into account.

To assess content validity (theoretical analysis), an expert panel consisting of three psychologists with clinical and research experience in the field of child psychology, psychiatry and psychotherapy were asked about: (1) the relevance of the tool and its items; (2) the clarity and age appropriateness of the items, and (3) the global feasibility of the questionnaire. Based on their comments, initial items of the preliminary tool were modified. Additionally, two parents of children aged 2 and 10 years were interviewed about whether the diction and phrasing was comprehensible and items seemed age-appropriate.

To provide an economical instrument, a pre-trial questionnaire was finally established with 28 items describing relationship-relevant child behaviors towards parents which had been rated by the aforementioned expert panel as being either positive or negative. To appropriately calculate a ratio, we aimed for equal numbers of questions on the valence scales (*n* = 14 positive, *n* = 14 negative behaviors). Items were designed to be rated on two scales following the structure of the Eyberg Child Behavior Inventory [32]. The Frequency Scale is meant to measure the frequency of relationship-relevant behaviors towards the parent from (1) *never* to (7) *always*, yielding an overall score of positive and negative behaviors, respectively. The second scale, the Problem Scale, was designed to allow the parent to provide a personal judgement as to whether or not she/he perceives the behavior to be “a problem” (“yes” or “no”). Responses on the Problem Scale were summed to create overall scores that indicate the degree to which the frequency and/or intensity of these positive and negative child behaviors, respectively, are viewed as problematic. This differentiation between the frequency of a specific relationship behavior and its evaluation as problematic or not has shown to be very helpful clinically as parents tend to differ in their tolerance for levels of child behavior. We assumed high frequencies of negative and low frequencies of positive relationship behaviors to be mostly perceived as problematic. Before we started the initial psychometric analysis of the pre-trial questionnaire, we expected to find:

(1) A two-factor solution for the pre-trial questionnaire with all items loading either on the positive or the negative child relationship behaviors factor.
(2) Good to excellent internal consistency for the Frequency and Problem Scales of both positive and negative child behaviors (i.e., *α*s/KR-20s ≥ 0.80, respectively).

#### 2.1.1. Procedures

An ethics committee approved the study which took place at a more rural German region (Schweinfurt). Participating parents were recruited from three day-care centers and one elementary school (1st to 4th grade) which had been personally informed about the purpose and content of this first study, before they decided whether to participate or not. Institutions delivered a set containing a cover letter and the pretrial version to the parents of each child enrolled from the age of two to ten years. All caregivers were asked to fill out questionnaires anonymously. Completed questionnaires were either returned in sealed envelopes without a name at the participating institutions or sent by postal services to the study site. Recruitment of parents took place from January 2012 until June 2012. To assess the suitability of the respondent data for factor analysis, Kaiser-Meyer-Olkin (KMO) Measure of Sampling Adequacy and Bartlett’s Test of Sphericity were used. Depending on the results of these measures, an exploratory factor analysis of the 28-item questionnaire (only Frequency Scale) was done. To avoid multiple parents reporting on the same child, only mother reports were used.

#### 2.1.2. Sample

The sample of this first study, a convenience sample, comprised 236 parents (mothers: *n* = 171, fathers: *n* = 65) reporting on 173 children (male: *n* = 87, female: *n* = 86) aged 2–10 years (*M*: 6.19 years, *SD*: 2.34 years). Besides child age and gender and parent gender, no other demographics were collected.

#### 2.1.3. Results

Based on a KMO index of 0.81 and a significant Bartlett’s Test of Sphericity (*p* ≤ 0.001), data were considered to be suitable for factor analysis. The scree test suggested two meaningful factors (percent variance: factor I: 24.17; factor II: 11.86). Thus, a two-factor solution was rotated using a varimax rotation. Principal axis factoring revealed that all items referring to positive child relationship behavior loaded positively on one factor (loading range: 0.48 to 0.77) whereas not all negative items loaded on the second factor. We found that only items describing the child actively showing a negative behavior (e.g., hitting the parent) loaded on the second factor whereas not showing a positive behavior (e.g., not asking the parent for help) did not. Additionally, high internal consistency was found for the Frequency Scale of positive behaviors (Cronbach’s α: 0.89) and the Problem Scales of positive (*KR-20*: 0.88) and negative behaviors (*KR-20*: 0.85), but not for the Frequency Scale of negative behaviors (Cronbach’s α: 0.73). The two factors correlated to a statistically significant, negative, and small degree (*r* = −0.30, *p* ≤ 0.001).

#### 2.1.4. Implications

The results of this pilot testing led to a change of eight items referring to relationship-relevant negative child behaviors. In order to emphasize the active role of the child, two of the items were verbally modified (e.g., “refuses me” instead of “refuses me when I am looking for physical contact”), and six new items replaced the previous ones (e.g., “provokes me“, and “makes fun of me” instead of “does not ask for my help if he/she is in trouble”, and “refuses to accept my praises and my recognition”). Another consequence was the decision to split the revised 28 items version into two independent, albeit related, Supplementary questionnaires: the Child Relationship Development Questionnaire (CRDQ; German: Fragebogen zur kindlichen Beziehungsgestaltung (FKB) [51], which assesses positive relationship-relevant child behavior, and the Child Relationship Checklist (CRC; German: Checkliste kindliches Beziehungsverhalten (CKB) [52]), which assesses negative child behaviors towards parents. This had two reasons: first, the two factors were not highly correlated (as intended with an orthogonal rotation). Furthermore, for economical reasons two independent questionnaires may be better suited to meet needs (and constraints) in clinical practice as questionnaires can be administered separately based on the requirements (e.g., assessment with the CRDQ if predominantly or exclusively positive relationship-relevant behaviors are of interest). CRDQ and CRC together constitute the Child Relationship Behavior Inventory (CRBI; German: Inventar zur Erfassung der kindlichen Beziehungsgestaltung [IEKB]). In line with Gottman and Levenson [48], we defined the CRBI balanced ratio as the ratio of the CRDQ Frequency Scale score (positivity) to the CRC Frequency Scale score (negativity).

### 2.2. Study 2: Assessing Parental Perceptions of Child Relationship Behaviors—The Child Relationship Behavior Inventory

Before we started the more comprehensive psychometric analysis of the inventory, we expected, based on the results from the pilot study, to find:

(1) A two-factor solution for the combined CRBI Frequency Scale with positive and negative child relationship behaviors.
(2) Good to excellent internal consistency for both the CRDQ’s and CRC’s Frequency and Problem Scales (i.e., *α*s and *KR-20*s ≥ 0.80, respectively).
(3) A low, negative association between the CRC and the CRDQ in terms of total frequency scores.
(4) Significantly higher frequency scores of positive relationship behaviors on mother ratings compared to father ratings. As mothers on average spend more time taking care of children [30], we assumed more emotional closeness between mother and child resulting in more positive child relationship behaviors.
(5) Significantly higher frequency scores of positive relationship behavior as well as significantly lower frequency scores of negative relationship behavior in girls compared to boys, and as a result of that higher CRBI ratios of girls compared to boys.
(6) A linear decrease of both positive and negative relationship behavior with the child’s age.
(7) A moderate positive association between externalizing symptoms and negative child relationship behaviors (as some negative child relationship behaviors might generalize to other contexts).
(8) Both significantly higher scores of positive child relationship behaviors and significantly lower scores of negative child relationship behaviors in children without (compared to those with) disruptive behavior problems.
(9) CRBI ratios to differentiate better than the CRBI scales between children with and without disruptive behavior problems (as the consequence of hypothesis 8).

#### 2.2.1. Procedure and Measures

An ethics committee approved the current study which took place at two German sites including a more rural region (Schweinfurt) and a university city (Braunschweig) across two states (Bavaria and Lower Saxony). Participating parents were recruited through day-care centers and elementary schools (1st to 4th grade). No specific exclusion criteria for participating institutions were defined. All institutions had been personally informed about the purpose and content of this study, before they decided whether to participate or not. Reasons for institutions to decline participation were: too many families with migration background and resulting language barriers and not enough resources due to participation in other studies.

Participating institutions delivered a set of study materials to the parents of each child enrolled from the age of two to ten years. Such a set contained a cover letter, the CRDQ, the CRC, and a special study questionnaire, which included 33 items assessing sociodemographic characteristics as well as medication and chronic physical and/or mental disorders. Sociodemographic information was obtained about children (age and gender) and each parent (e.g., age, gender, country of birth and educational level). Twenty-six of the 33 questionnaire items were taken from the KiGGS survey [53] (questions 1–12; 25; 46–47; 50–55; 84–88), a sociodemographically representative German sample. Socioeconomic status (SES) and migrant background were assessed according to the KiGGS study [54]. For SES, education, professional qualifications and status as well as net household income were taken into account. A child was classified as having migrant background, if both parents had been born outside Germany.

Additionally, all study packets included the *Eyberg Child Behavior Inventory (ECBI)* and two new, not yet validated questionnaires on child anxious feelings and mood (unpublished) and emotion regulation [55]. The ECBI is a widely used, validated parent rating scale designed to assess disruptive behavior of children aged 2 to 16 years [32]. It consists of 36 items, which are rated on two scales. Each item rating (on a scale of [1] *never* to [7] *always*) reflects the parental perception of behavior frequency (summed to obtain the Intensity Scale; range from 36 to 252) and whether this behavior is considered problematic or not (Problem Scale; YES/NO answer; range from 0–36). The Problem Scale is also considered an indicator of parental distress caused by child behavior. Psychometric evaluation of the German version of the ECBI derived from a community-based sample [56] showed high internal consistency for both scales on each age level (2 to 9 years) and good discrimination between different levels of behavior problems. Means of both the Intensity Scale and the Problem Scale were found to be significantly lower than in the U.S., resulting in recommended clinical problem behavior cut-off scores of 111 (Intensity Scale) and 12 (Problem Scale) [56]. The ECBI was both administered to investigate convergent (parental perception of negative child relationship behavior) and discriminant (parental perception of positive child relationship behavior) validity.

All caregivers were asked to fill out questionnaires anonymously. Completed questionnaires were either returned in sealed envelopes without a name at the participating institutions or sent by postal services to one of the two study sites. Questionnaires could only be linked to the recruiting institutions but not to children. Recruitment of parents took place from March 2014 until November 2015.

#### 2.2.2. Statistical Analyses

For data management and analysis we used SPSS 22, 23 and MPlus 7.11 [57], respectively. Unless otherwise stated, a two-sided significance level (*p* ≤ 0.05) was used for all statistical calculations. Bonferroni corrections were applied if more than one targeted comparison was conducted per hypothesis. Based on the large sample size of this study, we expected associations between scores to be detected as statistically significant, even if very small and in their meaning probably negligible. Thus, we decided to report both the significance of associations and their effect sizes which should guide us in the decision-making process.

##### Missing Data Management

If more than one item (>10%) on the CRDQ or the CRC Frequency Scale was missing, the whole questionnaire set of the caregiver was excluded from further analysis (*n* = 36; approximately 2% of all collected data). CRDQ as well as CRC Problem Scales were only included if both Frequency Scale and Problem Scale had not more than one item missing. A missing item was replaced by the mean score of the respective scale (CRDQ: Frequency Scale: 5; Problem Scale: 0; CRC: Frequency Scale: 2; Problem Scale: 0). This was the case in less than 5% of all items in these target questionnaires. ECBI scales for which more than four items were not completed were not included in further analysis. For up to four missing items the mean score of the scale was used to replace missing data [56].

##### Reliability

Reliability was estimated by internal consistency. Additionally, item selectivity was computed as part-whole corrected item-scale correlation. Items with correlations higher than 0.30 and lower than 0.70 were deemed acceptable [58]. We also investigated developmental dependencies of the construct. For this purpose, we defined four different age groups according to typical stages of child development: nursery school age (ages 2–4), kindergarten age (ages 5–6), early elementary school age (ages 7–8), and late elementary school age (ages 9–10). Linear contrasts were used to examine the hypothesis of a linear decrease of both positive and negative relationship behavior with the child’s age. Interrater-reliability was measured by intraclass correlations (ICC; two-way mixed model, absolute agreement). The following guidelines were used for interpretation: poor: ICC < 0.40; fair: ICC = 0.40–0.59; good: ICC = 0.60−0.74; excellent: ICC > 0.74 [59].

##### Validity

A confirmatory factor analysis (CFA) based on maximum likelihood estimation (ML) was conducted to determine whether the supposed two-factor structure of the CRBI provides an adequate representation of parent responses on the two questionnaires (construct validity). The adequacy of the factor structure was further tested by examining the CFA model across gender subgroups (i.e., boys and girls respectively). Only the dimension “Frequency” was examined (not the nominal “problem” scale (yes/no). While the χ^2^ statistic is reported for the present study, interpretation of model fit relied mainly on the incremental fit indices due to the frequent rejection of adequate models in large samples using χ^2^ [60].

Goodness of fit to the data for the resulting CRDQ/CRC factor model was evaluated using the Comparative Fit Index [CFI], the Root Mean Square Error of Approximation [RMSEA] and its 90% confidence interval (90% CI). Model fit is considered acceptable and good with CFI values larger than 0.900 and 0.950, respectively. RMSEA values of 0.05 or less reflect excellent model fit, values less than 0.10 reflect acceptable fit, while models with an RMSEA > 0.10 should be rejected [61]. The Standardized Root Mean Square Residual [SRMR] represents an absolute fit index and depicts the standardized difference between the observed correlation and the predicted correlation. A fit value of less than 0.08 is generally considered a good fit [62]. The CFA model assumed a two factor structure in which 14 items loaded onto a latent variable representing positive relationship-relevant child behaviors (i.e., the CRDQ Frequency Scale) and 14 items loaded onto a second latent variable representing negative relationship-relevant behaviors (i.e., the CRC Frequency Scale) respectively.

T-tests for dependent samples were used to analyze effects of child gender on Frequency and Problem Scale scores of mothers and fathers. We used linear contrasts to examine effects of age. As a prerequisite for the CFA, Mann-Whitney-U-Tests for independent samples were used to verify independence of observations for the CRBI frequency scales at the two data collection sites. Finally, perceived child relationship behavior was examined regarding its associations with child externalizing behaviors (ECBI). According to Cohen [63], a correlation coefficient of 0.10 is thought to represent a weak or small association; a correlation coefficient of 0.30 is considered a moderate correlation; and a correlation coefficient of 0.50 or larger is thought to represent a strong or large correlation. To analyze the discriminative validity of the CRC and the CRDQ, *t*-tests for independent samples were performed. We used the cut-off for the German ECBI Intensity Scale (raw score: 111, *t*-score 60) to discriminate between children with and without significant disruptive behavior problems. Moreover, to demonstrate which are the best and most significant predictors or discriminators of clinical versus non-clinical children (per ECBI scores) we assessed the incremental predictive validity with hierarchical logistic regression.

#### 2.2.3. Participants

Participating parents were recruited through 30 day-care centers (26 in the Schweinfurt area, and four in Braunschweig) and 25 elementary schools (1st to 4th grade) (23 in the Schweinfurt area, two in Braunschweig). A total of 5356 study packets was delivered to the participating institutions (4793 in the Schweinfurt area, 563 in Braunschweig) and given to the parents of all children aged two to ten years enrolled there. 1799 packets (1640 from the Schweinfurt area, 159 from Braunschweig) were returned. The mean response rate was 34% (28% from Braunschweig, 34% from Schweinfurt). Questionnaire sets referring to children younger than two years (*n* = 15), older than 10 years (*n* = 6) or without information about their age (*n* = 18) or sex (*n* = 2) were excluded from further analysis as well as invalid target questionnaires due to copying errors (CRDQ/CRC: *n* = 37) or questionnaires with no information about the informant’s relationship to the child (*n* = 9). In sum, 87 out of 1799 questionnaire sets (4.84%) were excluded. There was no significant difference between excluded and included sets in terms of socioeconomic status (χ^2^ = 5.1, *df* = 2, *p* = 0.08) or child gender (χ^2^ = 0.22, *df* = 1; *p* = 0.64). They also did not differ with regard to child (*t* = 0.004, *p* = 0.99) or parent age (mothers: *t* = −0.66, *p* = 0.51; fathers: *t* = −0.18, *p* = 0.86).

After managing for missing data (see above), the final sample for data analyses consisted of 1642 mother and 1068 father reports on 1712 children (32% of the addressed sample; 843 girls and 869 boys). In most cases both mother and father provided information on their child’s behavior (*n* = 999; 58.4%); for 643 children (37.6%), only mother reports could be included. A detailed sample description can be found in Table 1.

Mean child age was 6.61 (*SD* = 2.32) years. No significant sex differences were found (♀: *M* = 6.59 years; *SD* = 2.32 years; ♂: *M* = 6.63 years; *SD* = 2.32 years; *t*(1710) = 0.44, *p* = 0.66). At the time of our study, mothers’ mean age was significantly lower than the father’s mean age (*M_m_* = 37.11 years; *SD_m_* = 5.57 years; *M_f_* = 40.37 years; *SD_f_* = 6.48 years; *t*(1536) = 28.32, *p* ≤ 0.001; *d* = 0.54, medium effect). Significant correlations between the mother’s age and the maternal CRDQ Frequency Scale score (*r* = −0.069, *p* = 0.005) as well as the maternal CRC Problem Scale score (*r* = 0.096, *p* < 0.001) were found. In contrast, maternal CRDQ Problem Scale (*p* = 0.12) and CRC Frequency Scale scores (*p* = 0.17) did not correlate with the mother’s age. Similarly, there was a significant correlation between the father’s age and the paternal CRDQ Frequency score (*r* = −0.081, *p* = 0.008) and the paternal CRC Problem score (*r* = −0.075, *p* = 0.03) whereas CRDQ Problem Scale (*p* = 0.29) and CRC Frequency Scale (*p* = 0.83) did not correlate with the father’s age. All effect sizes were small (*r* < 0.10) indicating marginal clinical significance. Therefore, we did not control for the parent’s age in further analyses.

SES distribution in comparison to the KiGGS study on SES [64] showed a higher percentage of high compared to average SES (low SES: 19.4% vs. 20.0%, average SES: 52.4% vs. 60.0%, and high SES: 28.4% vs. 20.0%). The proportion of children with migration background in this study was slightly lower than in the KiGGS study (13% vs. 17%). Maternal Frequency Scale scores of the CRBI correlated significantly with SES (CRDQ: *r* = 0.077, *p* = 0.004; CRC: *r* = −0.064, *p* = 0.017). Fathers’ CRDQ Frequency scores were not significantly associated with SES (*r* = 0.052, *p* = 0.105) whereas CRC scores correlated significantly with SES (*r* = 0.165, *p* < 0.001). However, effect sizes were mostly small (*r* < 0.10), and even with small to moderate effects (*r:* 0.10 to 0.20, i.e., a shared variance of ≤4%), the clinical significance seemed marginal. Therefore, we did not control for SES in further analyses.

## 3. Results

### 3.1. Construct Validity (Hypothesis 1)

Mann-Whitney-U-Tests for independent samples revealed that maternal and paternal CRDQ/CRC scores were independent of the site at which data had been collected (CRDQ: mothers *z* = 0.333, *p* = 0.739; fathers: *z* = 0.621, *p* = 0.534; CRC: mothers: *z* = 0.560, *p* = 0.575; fathers: *z* = 1.777, *p* = 0.076).

All standardized factor loadings were significant for the two-factor model (*p* ≤ 0.05). The goodness of fit indices for each model are shown in Table 2. Absolute fit indices for the hypothesized two-factor model were mixed but similar for all tested subgroups. The RMSEA indicated acceptable model fit for all tested subgroups (0.072 ≤ RMSEA ≤ 0.081) with satisfactory 90% CI. The CFI did not meet recommended criteria for the assumed two-factor model in all subgroups (0.77 ≤ CFI ≤ 0.81), whereas the two-factor model met the SRMR criterion for good model fit (0.055 ≤ SRMR ≤ 0.067) in all subgroups.

### 3.2. Normative and Reliability Data (Hypothesis 2)

On a descriptive level, parents reported higher frequencies of positive compared to negative child behaviors: CRDQ Frequency Scale: Mothers: *M* = 76.98, *SD* = 10.46; Fathers: *M* = 70.34, *SD* = 12.20; CRC Frequency Scale: Mothers: *M* = 24.88, *SD* = 8.95; Fathers: *M* = 25.47, *SD* = 8.62. Behaviors on the CRC were more often evaluated as problematic than CRDQ behaviors (CRC Problem Scale: Mothers: *M* = 1.53, *SD* = 2.56; Fathers: *M* = 1.28, *SD* = 2.52; CRDQ Problem Scale: Mothers: *M* = 0.30, *SD* = 1.19; Fathers: *M* = 0.26, *SD* = 0.92). Moreover, parents entirely agreed on the most and the least frequent positive and negative child behaviors (CRDQ: “Shows me things that interest him/her.”, and “Spontaneously helps me with tasks.”; CRC: “Speaks to me in a commanding tone.”, and “Hurts me physically, e.g., biting …”). More detailed information (normative data for individual items) for both questionnaires can be found in Table 3 [CRDQ] and Table 4 [CRC]. Part-whole corrected item selectivity was acceptable [57] for all CRDQ and CRC items across different raters ranging from 0.40 to 0.69.

Mean scores, standard deviations and internal consistencies (Cronbach’s α) of positive relationship behavior are provided separately for boys and girls, different raters (mother vs. father) and age groups in Table 5. For corresponding data on negative relationship behaviors see Table 6. Internal consistency estimates for both scales of the CRC (frequency and problem) are high across different raters and age groups ranging from 0.80 to 0.91. Similarly, the CRDQ Frequency Scale demonstrates comparably high internal consistency across all age groups and raters (range: 0.85–0.90). The internal consistencies of the CRDQ Problem Scale range from 0.26 to 0.94 across ages with the average internal consistency of mother ratings on girls not falling within the acceptable range of 0.70 or higher. Table 5 also shows that boys reach higher mean values on the CRDQ Problem Scale than girls, and father ratings of girls, but not of boys, tend to show higher internal consistency than mother ratings.

Descriptive data (means and standard deviations) of the balance ratios for mothers and fathers can be found in Table 7. Across age groups and raters, the mean CRBI ratio score was 3.00–3.59 with slight variations across gender.

### 3.3. Independence of Positive and Negative Child Relationship Behaviors (Hypothesis 3)

Inter-correlations of CRDQ scales for both mother (*r* = −0.25, *p* < 0.001) and father ratings (*r* = −0.25, *p* < 0.001) indicate that there is a tendency of parents to consider low levels of positive relationship behavior as problematic (small effect). Additionally, positive inter-correlations of both CRC scales (mothers: *r* = 0.57, *p* < 0.001; fathers: *r* = 0.51, *p* < 0.001) suggest that higher frequency of negative relationship behavior is associated with a more negative parental evaluation (medium effect). A small and negative correlation could be found between the Frequency Scales of the CRDQ and the CRC (mothers: *r* = −0.25, *p* < 0.001; fathers: *r* = −0.25, *p* < 0.001). The Problem Scales of the CRDQ and the CRC showed a significantly positive inter-correlation (mothers: *r* = 0.28, *p* < 0.001; fathers: *r* = 0.22, *p* < 0.001; small effect). Thus, the scales share 5% to 8% of their variance.

### 3.4. Rater Effects (Hypothesis 4)

Some maternal CRBI scores correlated significantly with the mothers’ age (CRDQ Intensity Scale: *r* = −0.069, *p* = 0.005; CRC Problem Scale: *r* = −0.096; *p* ≤ 0.001), whereas others did not (CRDQ Problem Scale: *r*= −0.039, *p* = 0.120; CRC Intensity Scale: *r* = −0.035; *p* = 0.171). Similarly, some paternal CRBI scores correlated significantly with the fathers’ age (CRDQ Intensity Scale: *r* = −0.081, *p*= 0.008; CRC Problem Scale: *r* = −0.075, *p* = 0.026), whereas others did not (CRDQ Problem Scale: *r* = −0.033, *p* = 0.286; CRC Intensity Scale: *r* = 0.007, *p* = 0.824). As effects were trivial (*r* < 0.10), we did not control for the caregiver’s age in further analyses.

Regarding interrater reliability, CRDQ Frequency (ICC = 0.63) and Problem Scale scores (ICC = 0.66), CRC frequency (ICC = 0.73) and problem scores (ICC = 0.67) as well as CRBI ratios (*r* = 0.61, *p* < 0.001) indicated good correlations between mother and father ratings. As hypothesized a priori, significantly higher CRDQ frequency scores were found by means of t-tests for dependent samples for mother ratings compared with father ratings (*n* = 1011, *t*(1010) = 17.89, *p* < 0.001, *d* = 0.55). In contrast, no significant differences could be detected between mother and father ratings on the CRDQ Problem Scale (*n* = 973, *t*(972) = 0.79, *p* < 0.22, *d* = 0.03).

Regarding CRC frequency scores, we did not find any significant differences between raters (*n* = 848, *t*(847) = 1.14, *p* = 0.13, *d* = 0.04), but fathers evaluated negative relationship behaviors as less problematic than mothers (*n* = 804, *t*(803) = 3.38, *p* = 0.001, *d* = 0.12). Finally, we also found significant differences between maternal and paternal CRBI ratios with higher maternal scores (*n* = 829, *t*(828) = 7.56. *p* ≤ 0.001, *d* = 0.23, small effect). While some of these effects were statistically significant, their sizes were rather marginal.

### 3.5. Child Gender Effects (Hypothesis 5)

We had hypothesized a priori that CRDQ frequency scores should be higher and CRC frequency scores should be lower in girls compared to boys, which could be confirmed by one-sided tests. Both mother and father ratings on the CRDQ Frequency Scale were found to be significantly higher for girls when compared to boys (mothers: *t*(1641) = 6.12, *p* < 0.001, *d* = 0.30; fathers: *t*(1078) = 3.86, *p* < 0.001, *d* = 0.24), indicating small to medium effects. Additionally, mothers (*t*(1385) = 2.59, *p* < 0.01, *d* = 0.14) and fathers (*t*(999) = 1.71, *p* = 0.04, *d* = 0.11) reported significantly lower problem scores for girls (small effects).

Similarly, girls showed significantly lower CRC frequency scores on father ratings (*t*(931) = 1.76, *p* = 0.02, *d* = 0.11; small effect) but not on mother ratings (*t*(1578) = 1.48, *p* = 0.04, *d* = 0.08). CRC problem scales did not differ significantly between boys and girls (mothers: *t*(1498) = 0.77, *p* = 0.44, *d* = 0.04; fathers: *t*(894) = 1.64, *p* = 0.10, *d* = 0.11). CRBI ratios for girls were significantly higher than for boys both on mother (*t*(1567) = −3.36, *p* = 0.001, *d* = 0.17) and father ratings (*t*(911) = −2.68, *p* = 0.007, *d* = 0.18). While some of these effects were statistically significant, their sizes were rather small.

### 3.6. Child Age Effects (Hypothesis 6)

Linear contrasts were used to examine the hypothesis of a linear decrease of both positive and negative relationship behavior with the child’s age. CRDQ frequency scores did not correlate with the child’s age (mothers: *r* < 0.01, *p* = 0.95; fathers: *r* = 0.03, *p* = 0.42) nor could we find a decrease across age groups indicating a linear trend (mothers: *t*(1639) = 0.41, *p* = 0.34; fathers: *t*(1076) = 0.27, *p* = 0.40). However, CRDQ problem scores correlated significantly positively with the child’s age (mothers: *r* = 0.10, *p* < 0.01; fathers: *r* = 0.10, *p* < 0.01), indicating an increasing evaluation of negative child relationship behavior as problematic. Planned contrasts also showed linear increases in CRDQ problem scores across different age groups and raters (mothers: *t*(586) = 3.90, *p* < 0.01; fathers: *t*(490) = 2.88, *p* < 0.01). Effect sizes were small for mothers and fathers (*r*^2^ = 0.01).

In contrast, significantly negative correlations with the child’s age (mothers: *r* = −0.13, *p* < 0.01, fathers: *r* = −0.20, *p* < 0.01) as well as a linear decrease of CRC frequency scores across age groups were found for mother and father ratings (mothers: *t*(1576) = 5.99, *p* < 0.01; fathers: *t*(929) = 6.77, *p* < 0.01). CRC problem scores correlated negatively with the child’s age (mothers: *r* = −0.05, *p* = 0.04; fathers: *r* = −0.07, *p* = 0.03), and significant planned contrasts validated these results (mothers: *t*(1009) = 2.45, *p* < 0.01; fathers: *t*(652) = 1.94, *p* = 0.03). However, all reported effects were small (*r*^2^_(max)_ = 0.04).

Thus, as we had expected, the frequency of problematic child relationship behavior significantly decreased with increasing child age whereas – contrary to our a priori hypothesis—positive relationship-relevant child behavior did not show any correlation with the child’s age. With regard to CRBI ratios, we found small to moderate age effects both on mother (ηp2 = 0.035) and father ratings (ηp2 = 0.05). In contrast to our a priori hypothesis, we found no linear decrease of CRBI ratios across ages, but an increase. No significant interaction between age and gender could be found. While some of these effects were statistically significant, their sizes were rather trivial.

### 3.7. Criterion Validity (Hypothesis 7)

Table 8 summarizes correlations between the CRBI questionnaires and the scales of the ECBI (mother and father ratings). The consistent, statistically significant negative correlations between CRDQ frequency and ECBI intensity scores support the divergent validity of the CRDQ. Concordant with our hypothesis, moderate to large associations were found between CRC and ECBI scales. These results support the concurrent as well as convergent validity of the ECBI and CRC.

### 3.8. Disruptive Behavior Problems and Perceived Child Relationship Behavior (Hypothesis 8)

As expected, we found significantly higher scores of positive child relationship behaviors and significantly lower scores of negative child relationship behaviors for children without disruptive behavior problems compared to children with such problems. For mother and father ratings all scales of the CRBI showed significant discriminative validity with lowest effect sizes for the CRDQ Problem Scale and highest effect sizes for the CRC Frequency Scale (see Table 9).

CRBI ratios on mother and father ratings significantly differed between children who met the ECBI Intensity Scale cutoff (≥111) for the presence of disruptive behavior problems (mothers: *M* = 2.33, *SD* = 0.77; fathers: *M* = 2.12, *SD* = 0.73) and those who did not (mothers: *M* = 3.78, *SD* = 1.22), *t*(755) = −26.07; *p* < 0.001; fathers: *M* = 3.41, *SD* = 1.22, *t*(616) = −18.96; *p* < 0.001) with effect sizes (Cohen’s *d*) above 1.

Additionally, we performed two logistic regressions, one for mothers and one for fathers, with clinical versus non-clinical children (per ECBI scores) to determine the best and most significant predictors of this group assignment. The primary focus was on assessing the incremental predictive validity of the CRBI ratios (compared to the single variables; entered as predictors in the first model) using hierarchical logistic regression.

Mothers: The omnibus tests of model coefficient for model 1 (maternal intensity and problem scores) yielded a χ^2^ (4) of 408.26 (*p* < 0.0001) indicating that the model with these scores as predictors is better than one that has no predictors. The Wald statistic was significantly different for maternal intensity scores (CRDQ: *β* = −0.03, *p* < 0.001; CRC: *β* = 0.14, *p* < 0.001), but not for problem scores. Thus, higher frequency scores on the CRDQ predicted lower probability to show significant disruptive behavior problems (OR = 0.97), while for CRC frequency scores the situation was precisely reverse: Higher scores predicted higher probability to be in the clinical group (OR = 1.16). In model 2, we added the ratio. This model still showed a significant data fit (χ^2^ (5) = 426.70, *p* < 0.001). Furthermore, model 2 demonstrated significant improvement compared to model 1 (χ^2^ (1) = 18.44, *p* < 0.001). It could be shown that the CRBI ratio is a significant predictor (*β* = −1.22, *p* < 0.001) with an OR of 0.295, indicating that the probability to be in the clinical group is lower with an increasing ratio. All other predictors were non-significant except the CRDQ problem score which now also yielded a significant regression coefficient (*β* = 0.137, χ^2^ (1) = 4.93, *p* = 0.26, OR = 1.15). This indicates that in addition to the CRBI ratio only the maternal evaluation of positive child relationship behavior as problematic was of further relevance when all predictors in model 2 are considered.

Fathers: The omnibus tests of model coefficient for model 1 (paternal intensity and problem scores) yielded a χ^2^ (4) of 262.53 (*p* < 0.001), indicating that the model with these scores as predictors is better than one that has no predictors. The Wald statistic was significantly different for paternal intensity scores (CRDQ: *β* = −0.03, *p*= 0.005, OR = 0.975; CRC: *β* = 0.15, *p* < 0.001, OR = 1.17), but not for problem scores. Thus, in this first model, results for fathers were very similar to the results for mothers. For model 2, we added the ratio, and this model still showed significant data fit (χ^2^ (5) = 264.06, *p*< 0.001). However, model 2 did not demonstrate a significant improvement compared to model 1 (χ^2^ (1) = 1.53, *p* = 0.217). Thus, the CRBI ratio was not a significant predictor in fathers (*β* = −0.458, *p* = 0.23; OR = 0.63). Except the CRC intensity score (*β* = 0.112, *p* = 0.002, OR = 1.118), all other predictors were non-significant.

To sum up, the results of hierarchical logistic regression indicate that, in contrast to mothers, the paternal CRBI ratio is not incrementally valid in the prediction of clinical group membership. For fathers, the more central predictor in this equation was the perceived frequency of negative child relationship behavior (i.e., the CRC Frequency Scale).

## 4. Discussion

The CRBI, a new tool consisting of two 14-item questionnaires, has been designed to measure the parental perception of negative and positive child behaviors towards parents; it does not assess the perceived or actual function of child behaviors. Psychometric evaluation of the CRDQ and the CRC clearly supported the theoretically based assumption that positive and negative child behaviors as perceived by their parents represent two mostly independent aspects of child relationship behavior towards parents. CFA revealed an overall acceptable fit of the hypothetically underlying latent variables of the CRBI (i.e., positive and negative child relationship behaviors) with the sample data. While absolute fit indices (RMSEA and SRMR) showed good to acceptable fit, the CFI, representing an incremental or relative fit index, did not meet criteria for adequate model fit. However, as the a priori model of two factors was set, no modifications were performed in order to improve fit indices. Additionally, for the CRDQ Frequency Scale, divergent validity could be demonstrated by means of a comparison with the ECBI Intensity Scale. In contrast, CRC scales share about 44% to 50% of their variance with the corresponding ECBI scales, demonstrating concurrent as well as convergent validity. While the ECBI comprises disruptive behaviors towards parents, siblings, other adults and peers, and the CRC exclusively measures negative relationship behaviors towards parents, our results indicate that the parents’ perception of the overall child behavior is significantly influenced by their perception of their child’s behaviors towards themselves. Bearing this in mind, the decision whether to apply a measure like the ECBI or the CRC primarily depends on the focus of the psychological evaluation.

Frequency scales of the CRDQ and the CRC as well as the CRC Problem Scale showed very good internal consistencies for both mother and father ratings and across all age groups suggesting that they are homogeneous measures of perceived child relationship behavior. In contrast, the CRDQ Problem Scale failed to achieve acceptable internal consistency across all age groups, especially for girls. We suppose this is due to very restricted variances in CRDQ problem scores, especially for girls. However, the scale may still be important in specific contexts, e.g., when children with very low (or overly high) levels of inappropriately expressed positive relationship behavior (e.g., in order to manipulate a parent) are the focus of research and practice (e.g., children with certain types of attachment disorders or autism). For the general population or clinical samples with rather high levels of positive behavior, it might be better in the future to ask parents whether they are satisfied with the child’s positive behavior or not. Moreover, such wording might be more consistent with a scale on positive behaviors. Last but not least, a second CRBI ratio composed of problem and satisfaction scores could be a more balanced measure for parental stress than for example the ECBI Problem Scale score.

With regard to parent gender effects, mothers reported more positive child relationship behaviors towards them while fathers reported fewer problems with negative relationship behavior than mothers. There are several possible explanations for these findings. With regard to more positive child behaviors towards mothers, the amount of time spent together with the child and the quality of the interaction may be crucial. Mothers on average do not only spend more time overall with their children [65], but more time taking care of children, even when mothers work full time [66]. Moreover, mothers have been repeatedly found to use more supportive speech like praise with their children than fathers [15,23]. Thus one might suppose that a child would feel more emotional closeness to his/her mother (who in most cases is the child’s primary caregiver) resulting in more positive child relationship behaviors. Another reason may be grounded in traditional gender-dependent parental role expectations and attitudes (mothers as the primary caretakers providing the loving care and support needed by children; fathers as the breadwinners setting clear limits) [67]. However, even if mothers are so far the primary caregivers of most children, this issue should be opened to current social changes in family settings (e.g., same-sex parenting, fathers being primary caregivers). The finding that in this study fathers reported fewer problems with negative child behaviors than mothers is in line with other studies [18,20]. Again, one possible explanation might be that mothers spend more time with their children than fathers, thus having more opportunities to experience difficulties with their children [20]. Another reason could be that children are more compliant with fathers than mothers resulting in lower paternal problem scores [15,18]. This different interparental problem perception might also explain why mothers are far more likely than fathers to seek professional help [15,68].

Interestingly, our study results clearly indicate child gender effects. However, most effect sizes have been small. In general, girls are perceived by their parents in a more positive way than boys (higher scores on the CRDQ Frequency Scale and at least a tendency to lower CRC frequency scores as well as higher CRBI ratios). These findings are in line with studies showing that compared to males, females aged 3 to 12 years are more concerned about sharing, turn-taking and cooperation, and that they are more prosocial from childhood through adolescence [11,12]. Moreover, they are concordant with studies which found boys to present more disruptive behavior problems than girls during childhood and adolescence [13,14]. A reason for or a consequence of these findings might be that mothers talk more and use more supportive speech with daughters than with sons [23]. However, our findings might also reflect gender stereotypes with parents attributing more positive dyadic relationship behavior to girls compared to boys.

As we had expected, the frequency of problematic child relationship behavior significantly decreased with increasing child age, which is in line with studies in the general population showing an age-dependent decrease of externalizing behaviors [69]. In contrast, positive relationship-relevant child behavior did not show any correlation with the child’s age, and CRBI ratios increased across age groups indicating a trend towards more positivity. To our best knowledge, these are new findings which emphasize the importance of assessing both positive and negative child relationship behaviors during the general psychological assessment process. Moreover, they suggest an overall development towards relatively more positivity in the parent-child relationship (as perceived by the parent) from the age of two to the age of ten.

Last, but not least, scales of the CRDQ and the CRC as well as the CRBI ratio demonstrated good validity for discriminating between children with and without significant disruptive behavior problems as indicated by the ECBI. Moreover, the results of hierarchical logistic regression indicated that, in contrast to fathers, the CRBI ratio of mothers is incrementally valid in the prediction of significant disruptive child behavior problems as we had hypothesized a priori. For fathers, the more central predictor in this equation was the perceived frequency of negative child relationship behavior (i.e., the score of the CRC Frequency Scale). These results suggest that across raters different aspects covered by the CRBI (e.g., CRBI ratio versus CRC Frequency and Problem Scale scores) might be of different relevance for a) the discrimination between children with or without a specific disorder (e.g., autism spectrum disorder), and b) the differentiation between different diagnostic groups (e.g., internalizing versus externalizing disorders). To test these hypotheses, studies on the discriminative power of the CRBI are needed, especially in clinical populations of children aged 2–10 years.

This study also reveals some interesting findings with regard to the parental perception of child relationship behavior in the general population. We found good interrater-reliability for both positive and negative child relationship behaviors whereas meta-analyses on interparental agreement of child behavior problems [70] and social competence of children [71] reported only moderate correspondences. Across gender, broad consensus could be demonstrated among parents on the most frequent positive (“Shows me things that interest him/her”) and negative (“Speaks to me in a commanding tone“) child behaviors towards them. Parents also shared the perception that “Spontaneously helps me with tasks” and “Hurts me physically [e.g., biting, …]” occur least often. As expected, significantly higher frequencies of positive compared to negative relationship behavior were found in the general population resulting in mean CRBI ratio scores for boys and girls ranging from 3.00 to 3.59. This ratio as a balanced measure of positive and negative relationship-relevant behaviors might be a good measure of the overall parent-child relationship quality (from the parent’s perspective). Parent-child relationship quality is clearly related to the development and generalization of child psychosocial competence and psychopathology, e.g., [71,72], and it can be seen as an important factor for the emergence of positive or negative developmental cascades causing or moderating positive or negative trajectories across functions and domains [73]. Therefore, the CRBI as a balanced measure of parent-child relationship quality could be helpful for use in both clinical and healthy samples in order to figure out how parent and child behaviors uniquely, bi-directionally, and synergistically create positive or negative developmental cascades.

Interestingly, for couple relationships, Gottman [46] found a ratio of 5:1 (positivity to negativity) to be typical for stable marriages while 0.8:1 was indicative of unstable marriages. As the present study is cross-sectional, we can only relate our results to the difference in ratios between groups of children with and without significant disruptive behaviors: While the ratio of the first group approaches about two positives to one negative, the other is displaying a ratio of about 3 to 3.5:1. Analogous to the findings of Gottman and colleagues [47] that the ratio model predicts marriage stability, one might suggest that the CRBI ratio is of significance for parent-child relationship stability in general. Moreover, it could be predictive of many key issues in parent-child relationship (e.g., parental burn-out, child abuse and neglect). Longitudinal studies are needed to investigate these hypotheses.

This study comprises a large sample of more than 1700 children aged 2 to 10 years from two different sites in two different states of Germany, representing a satisfactory response rate of 34% of all addressed families. However, it is a self-selected sample. As the survey was carried out anonymously we do not have any information about families who refused to participate. Furthermore, a comparison of our sample to the most recent sociodemographically representative sample available for Germany, the KiGGS survey [64], reveals a slightly different composition of our sample with regard to socioeconomic status (more families of high SES and less families of medium SES than in the KiGGS survey). There are some more limitations that should be addressed. First, validity of the CRBI was only assessed with the ECBI but not with measures of parental perceptions or cognition, such as parental attributions, parental self-efficacy or parenting satisfaction although this would have been a good idea. The reasons for not including such measures were that we did not want to further increase the number of questions, and that the behaviorally-focused ECBI seemed to be a better choice for examining both convergent and divergent validity. Second, CRBI scores were not validated against objective behavioral observations as this would have been beyond the scope of this study. However, such a validation should be done in the future.

This study has several strengths, among them item generation based on the combination of both deductive and inductive approaches (as suggested by e.g., [74]), a relatively large pilot study, well-documented response rates across participating institutions, the combined use of exploratory and confirmatory factor analysis techniques [74], a very large main sample, and the inclusion of both mother and father reports on almost 1000 children, which allows an excellent comparison between the parents’ perceptions of their children. This sample size also allowed similarly large age groups of children.

## 5. Conclusions

Our results suggest that the CRBI is a valid parent-report measure, which, in contrast to most other child assessment measures like the ECBI, does not exclusively focus on child behavior problems but equally includes competent child behaviors in the context of parent-child relationship thus providing a balanced understanding of perceived child behavior in the parent-child relationship, especially when using the CRBI ratio. So far, such a measure has been missing although it could be very useful for clinical and research purposes given the importance of parent-child relationship quality for child outcomes and mental well-being over the lifespan [8]. To assess both positive and negative child relationship behaviors could help improve our understanding of the relevance of these different aspects for the development of the parent-child relationship. Furthermore, parent-child relationship quality is dynamic, and its course may be observed by repeatedly assessing this ratio. Interventions like parent management programs or individual therapy may also use the CRBI to assess effects in changing both positive and negative child relationship behaviors. Thus, the potential value of the CRBI ranges from general child development to intervention science.

Next steps will include the assessment of test-retest reliability, of the instrument’s psychometric properties among clinical cases, of its usefulness for discriminating clinical and non-clinical cases of relationship problems (e.g., parent-child relationship of children with attachment disorders and their primary caregivers), of its potential to discriminate between different diagnostic groups, and of its responsiveness to change in intervention studies (e.g., parent management programs like Parent-Child Interaction Therapy [75]). Furthermore, next steps will also include the translation of the CRBI into different languages, and its cross-cultural adaptation and validation. This work is partially already underway (English and French versions available).

## Figures and Tables

**Table 1:** Demographic characteristics of the study sample.

		*N*	Percent
Children sample		1712	100.0
Sex	Female	843	49.2
	Male	869	50.8
Child lives with	Both parents	1412	82.6
	Single mother	171	10.0
	Mother and new partner	89	5.2
	Single father	11	0.6
	Foster parents	17	1.0
	Father and new partner	7	0.4
	Adoptive parents	2	0.1
	Missing	3	---
Migration background ^1^	Yes	223	13.0
	No	1489	87.0
Parent sample		2710	100.0
Sex	Female	1642	60.1
	Male	1068	39.9
Socioeconomic	Low	292	19.4
status ^2^	Medium	787	52.4
	High	424	28.2
	Missing	209	---

^1^ Migrant background was also assessed according to the KiGGS study [54], taking into account the parents’ country of birth. A child was classified as a migrant, if both parents had been born outside Germany.; ^2^ Socioeconomic status (SES) was assessed according to the KiGGS study [54], taking into account education, professional qualifications and status as well as net household income.

**Table 2:** Model fit statistics for the tested two-factorial model of the CRDQ/CRC scales for all subgroup analyses with mother and father ratings of child behavior.

	*N*	*df*	χ^2 1^	RMSEA ^2^	RMSEA 90% CI ^2^	SRMR ^3^	CFI ^4^
**Mothers**	1579	349	3613.06 ***	0.077	0.075–0.079	0.058	0.79
**girls**	768	349	1894.14 ***	0.076	0.073–0.079	0.055	0.79
**boys**	811	349	2201.47 ***	0.081	0.078–0.084	0.067	0.76
**Fathers**	921	349	2204.31 ***	0.076	0.073–0.079	0.058	0.8
**girls**	447	349	1280.31 ***	0.077	0.073–0.082	0.061	0.81
**boys**	474	349	1358.54 ***	0.078	0.074–0.083	0.065	0.79

^1^ Chi square test of model fit and its associated degrees of freedom (*df*). ^2^ Root Mean Square Error of Approximation and its 90% Confidence Interval. ^3^ Standardized Root Mean square Residual. ^4^ Comparative Fit Index.; *** *p* < 0.001.

**Table 3:** Normative data for individual items of the Child Relationship Development Inventory (CRDQ) on mother and father ratings.

		Mothers				Fathers			
			Frequency		Problem		Frequency		Problem
Item #	Item Content (In Part Abbreviated)	Item Selectivity ^1^	*M*	*SD*	%	*Item Selectivity* ^1^	*M*	*SD*	%
01.	Gives me small presents (e.g., drawings).	0.51	5.04	1.29	1	0.54	4.62	1.32	0
02.	Praises or compliments me.	0.63	4.74	1.32	1	0.69	4.40	1.37	0
03.	Tells me that he/she likes me.	0.64	5.66	1.30	1	0.68	5.18	1.45	2
04.	Gives me a kiss.	0.53	5.83	1.35	1	0.58	5.22	1.64	1
05.	Hugs or caresses me/cuddles with me.	0.57	5.98	1.01	1	0.61	5.46	1.25	1
06.	Shows me things that interest him/her..	0.47	6.11	0.84	1	0.49	5.80	0.94	1
07.	Tells me what makes him/her happy.	0.58	5.92	1.06	2	0.60	5.55	1.15	1
08.	Tells me what stresses him/her.	0.58	5.20	1.40	9	0.60	4.57	1.44	9
09.	Approaches me to spend time together.	0.50	5.73	1.02	2	0.50	5.57	1.06	1
10.	Approaches me to be comforted.	0.49	5.87	1.16	2	0.61	4.98	1.31	2
11.	Shares with me (e.g., food).	0.47	5.26	1.26	1	0.50	4.96	1.32	1
12.	Tries to comfort me if I feel bad.	0.58	5.37	1.41	2	0.68	4.57	1.61	1
13.	Spontaneously helps me with tasks.	0.54	4.48	1.39	5	0.55	4.14	1.41	5
14.	Talks about me in a good manner.	0.51	5.59	1.31	1	0.52	5.28	1.42	1
Item mean		0.54	5.48	1.22	2.14	0.58	5.02	1.34	1.86
Total Scale mean			76.98	10.46			70.34	12.20	

^1^ Part-whole corrected.

**Table 4:** Normative data for individual items of the Child Relationship Checklist (CRC) on mother and father ratings.

		Mothers				Fathers			
			Frequency		Problem		Frequency		Problem
Item #	Item Content (In Part Abbreviated)	Item Selectivity ^1^	*M*	*SD*	%	Item Selectivity ^1^	*M*	*SD*	%
01.	Speaks to me in a commanding tone.	0.62	2.82	1.41	25	0.57	2.72	1.40	17
02.	Insults me.	0.67	1.82	1.07	16	0.67	1.83	1.04	13
03.	Ignores me on purpose.	0.56	1.80	1.09	12	0.50	1.96	1.11	11
04.	Tells me what to do.	0.66	2.05	1.23	11	0.65	2.12	1.20	9
05.	Threatens me.	0.63	1.36	0.80	6	0.60	1.41	0.78	6
06.	Provokes me.	0.68	2.42	1.40	21	0.64	2.26	1.30	14
07.	Criticizes me in a derogatory way.	0.58	1.33	0.76	6	0.56	1.35	0.67	4
08.	Disturbs me on purpose when I need a rest.	0.56	2.07	1.19	13	0.55	2.06	1.17	11
09.	Makes fun of me.	0.43	1.34	0.76	3	0.42	1.44	0.79	2
10.	Complains about me.	0.54	1.54	0.87	4	0.49	1.59	0.82	3
11.	Tells me that he/she doesn’t like me.	0.41	1.45	1.03	5	0.41	1.56	1.00	6
12.	Lies to me.	0.43	2.07	1.08	18	0.40	2.04	1.04	15
13.	Rejects me.	0.51	1.57	0.80	7	0.52	1.79	0.94	8
14.	Hurts me physically (e.g., biting, …)	0.45	1.23	0.65	7	0.47	1.27	0.69	6
Item mean		0.55	1.78	1.01	11.00	0.53	1.81	1.00	8.93
Total scale mean			24.88	8.95			25.47	8.62	

^1^ Part-whole corrected.

**Table 5:** Normative data (means and standard deviations) and internal consistency (*α* or KR-20) of the Child Relationship Development Inventory (CRDQ) for different raters and age-groups.

CRDQ	MOTHERS					FATHERS				
AGE GROUPS	*n* f/m ^1^	Problem		Frequency		*n* f/m ^1^	Problem		Frequency	
		*M (SD)* f/m ^1^	*KR-*20 f/m ^1^	*M (SD)* f/m ^1^	*α* f/m ^1^		*M (SD)* f/m ^1^	*KR-*20 f/m ^1^	*M (SD)* f/m ^1^	*α* f/m^1^
Ages 2–4	185/187	0.11/0.13 (0.42)/(0.58)	0.47/0.69	78.21/74.49 (10.02)/(10.24)	0.87/0.85	139/130	0.05/0.13 (0.31)/(0.43)	0.49/0.37	69.60/68.32 (13.09)/(11.48)	0.91/0.88
Ages 5–6	162/177	0.19/0.34 (0.50)/(1.15)	0.26/0.81	79.20/76.54 (10.29)/(10.27)	0.88/0.88	118/122	0.25/0.37 (0.66)/(1.25)	0.45/0.84	73.75/69.95 (10.74)/(10.95)	0.89/0.87
Ages 7–8	267/252	0.20/0.40 (0.75)/(1.31)	0.31/0.84	79.52/75.52 (9.12)/(10.65)	0.88/0.87	154/170	0.29/0.34 (1.17)/(0.88)	0.87/0.65	72.68/69.05 (11.51)/(13.27)	0.90/0.90
Ages 9–10	190/222	0.39/0.58 (1.48)/(2.04)	0.67/0.94	77.06/75.30 (11.11)/(11.00)	0.89/0.88	116/131	0.26/0.40 (0.68)/(1.35)	0.33/0.86	71.29/68.52 (12.48)/(12.53)	0.90/0.90
Total	804/838	0.22/0.38 (0.89)/(1.41)	0.51/0.89	78.57/75.45 (10.09)/(10.58)	0.88/0.87	527/553	0.21/0.31 (0.80)/(1.03)	0.74/0.79	71.80/68.95 (12.07)/(12.18)	0.90/0.89

^1^ f = female; m = male.

**Table 6:** Normative data (means and standard deviations) and internal consistency (α or KR-20) of the Child Relationship Development Inventory (CRC) for different raters and age-groups.

CRC	MOTHERS					FATHERS				
AGE GROUPS	*n* f/m ^1^	Problem		Frequency		*n* f/m ^1^	Problem		Frequency	
		*M (SD)* f/m ^1^	*KR-20* f/m ^1^	*M (SD)* f/m ^1^	*α* f/m ^1^		*M (SD)* f/m ^1^	*KR-20* f/m ^1^	*M (SD)* f/m ^1^	*α* f/m^1^
Ages 2–4	176/181	1.68/1.84 (2.52)/(2.97)	0.84/0.87	25.99/27.78 (8.43)/(9.75)	0.85/0.88	133/113	1.37/1.84 (2.58)/(2.97)	0.87/0.88	27.29/29.43 (8.41)/(8.87)	0.84/0.84
Ages 5–6	156/176	1.64/1.35 (2.70)/(2.37)	0.88/0.86	25.53/26.38 (8.14)/(8.41)	0.86/0.84	95/103	1.02/1.35 (1.89)/(2.37)	0.80/0.85	26.32/26.39 (8.48)/(9.33)	0.85/0.88
Ages 7–8	254/246	1.26/1.22 (2.42)/(2.93)	0.88/0.88	23.56/23.47 (8.25)/(8.28)	0.88/0.88	122/143	1.28/1.22 (2.54)/(2.93)	0.89/0.92	22.90/24.38 (6.60)/(8.38)	0.81/0.89
Ages 9–10	183/208	1.42/1.29 (2.43)/(2.52)	0.85/0.87	23.63/24.00 (9.94)/(9.48)	0.91/0.90	104/120	0.78/1.29 (1.73)/(2.52)	0.82/0.89	23.16/24.18 (8.90)/(8.22)	0.90/0.88
Total	769/811	1.47/1.42 (2.51)/(2.73)	0.86/0.87	24.53/25.20 (8.75)/(9.12)	0.88/0.88	454/479	1.14/1.42 (2.27)/(2.73)	0.87/0.89	24.96/25.95 (8.31)/(8.90)	0.86/0.88

^1^ f = female; m = male.

**Table 7:** Descriptive data (means and standard deviations) of the CRBI ratio (positivity: negativity) for boys and girls across different raters (1,569 mothers and 913 fathers) and age-groups.

	Mothers			Fathers		
	N	CRBI Ratio *M* (*SD*)		N	CRBI Ratio *M* (*SD*)	
Age Groups	Girls/Boys	Girls	Boys	Girls/Boys	Girls	Boys
Ages 2–4	176/181	3.31 (1.11)	2.97 (0.99)	131/113	2.82 (1.10)	2.57 (1.01)
Ages 5–6	155/176	3.38 (1.09)	3.22 (1.17)	94/102	3.14 (1.20)	2.97 (1.24)
Ages 7–8	251/242	3.77 (1.30)	3.62 (1.32)	120/139	3.49 (1.23)	3.26 (1.21)
Ages 9–10	181/207	3.79 (1.52)	3.58 (1.36)	98/116	3.52 (1.43)	3.26 (1.31)
Total	763/806	3.59 (1.29)	3.37 (1.26)	443/470	3.22 (1.27)	3.00 (1.23)

**Table 8:** Correlation of the CRBI questionnaires with the ECBI (Frequency and problem scales).

MOTHERS	ECBI Intensity Scale	ECBI Problem Scale	FATHERS	ECBI Intensity Scale	ECBI Problem Scale
***CRDQ***			***CRDQ***		
Frequency Scale	−0.32 **	−0.20 **	Frequency Scale	−0.31 **	−0.23 **
Problem Scale	0.18 **	0.32 **	Problem Scale	0.21 **	0.29 **
***CRC***			***CRC***		
Frequency Scale	0.71 **	0.46 **	Frequency Scale	0.66 **	0.41 **
Problem Scale	0.44 **	0.71 **	Problem Scale	0.41 **	0.71 **

** *p* ≤ 0.01.

**Table 9:** Discriminant validity of the CRBI scales and the CRBI ratio.

**Scale/Ratio**	***Mother Ratings***					
	**ECBI Intensity** **Score ≥ 111**		**ECBI Intensity** **Score < 111**		***t (df)***	***d***
	***N***	***M (SD)***	***N***	***M (SD)***		
CRDQ Frequency	329	72.88 (9.96)	1257	78.08 (10.20)	−8.27 (1584) ***	0.52
CRDQ Problem	322	0.63 (1.53)	1220	0.19 (0.94)	4.88 (386) ***	0.34
CRC Frequency	315	34.20 (10.70)	1217	22.51 (6.64)	18.50 (378) ***	1.31
CRC Problem	303	3.25 (3.35)	1152	1.05 (2.08)	10.87 (364) ***	0.79
CRBI Ratio	314	2.33 (0.77)	1210	3.78 (1.22)	−26.07 (755) ***	1.42
**Scale/Ratio**	***Father Ratings***					
	**ECBI Intensity** **Score ≥ 111**		**ECBI Intensity** **Score < 111**		***t (df)***	***d***
	***N***	***M (SD)***	***N***	***M (SD)***		
CRDQ Frequency	257	65.56 (11.08)	790	71.69 (12.23)	−7.14 (1045) ***	0.53
CRDQ Problem	252	0.49 (1.19)	758	0.18 (0.70)	4.00 (310) ***	0.33
CRC Frequency	220	33.39 (8.86)	684	22.94 (6.77)	16.05 (305) ***	1.33
CRC Problem	215	2.73 (3.56)	650	0.81 (1.85)	7.59 (253) ***	0.68
CRBI Ratio	217	2.12 (0.73)	676	3.41 (1.22)	−18.96 (616) ***	1.28

*** *p* ≤ 0.001.

## Supplementary Materials

The following are available online at https://www.mdpi.com/1660-4601/16/7/1123/s1, Questionnaire S1: Child Relationship Development Questionnaire (CRDQ), Questionnaire S2: Child Relationship Checklist (CRC).
